# Three-Dimensional Nickel Foam-Based Lithiophilic LPP-Ni_3_S_2_@Ni Current Collector for Dendrite-Free Lithium Anode

**DOI:** 10.3390/nano14131158

**Published:** 2024-07-07

**Authors:** Xin Zhang, Linli Guo, Sheng Huang, Dongmei Han

**Affiliations:** 1 School of Chemical Engineering and Technology, Sun Yat-sen University, Guangzhou 510275, China; zhangx349@mail2.sysu.edu.cn (X.Z.); guolli3@mail2.sysu.edu.cn (L.G.); 2 Key Laboratory of Low-Carbon Chemistry & Energy Conservation of Guangdong Province/State Key Laboratory of Optoelectronic Materials and Technologies, School of Materials Science and Engineering, Sun Yat-sen University, Guangzhou 510275, China; huangsh47@mail.sysu.edu.cn

**Keywords:** nickel foam, lithium metal anode, lithiophilic interface layer, nucleation

## Abstract

Lithium metal has been treated as one of the most promising anode materials for next-generation rechargeable batteries due to its extremely high theoretical capacity. However, its practical application is hindered by inhomogeneous lithium deposition and uncontrolled dendrite growth. In this work, we prepared a three-dimensional nickel foam (NF)-based current collector with a lithiophilic interface layer through facile hydrothermal and coating methods. The lithiophilic Ni_3_S_2_ array synthesized via a hydrothermal method has been demonstrated to facilitate the nucleation of Li^+^. Moreover, it has been observed that the outer coating comprising LPP effectively enhances the inward diffusion of Li^+^. Additionally, this interface layer can serve as an isolating barrier between the electrodes and the electrolyte. The prepared LPP-Ni_3_S_2_@Ni shows significant reversibility both in symmetric cells (1200 h, 1 mA cm^−2^) and half-cells (CE: 99.60%, 500 cycles, 1 mA cm^−2^) with low interfacial resistance (35 Ω). Full cells with LiFePO_4_ as a cathode also exhibit promising electrochemical performance with over 76.78% capacity retention over 200 cycles at 1 C.

## 1. Introduction

To meet rapid development in portable electronics and electric vehicles, demand for energy storage devices becomes urgent. Lithium metal batteries, which select lithium metal as an anode, promise high energy densities for rechargeable batteries. Lithium metal is one of the most ideal anode materials due to its ultrahigh theoretical specific capacity (3860 mAh g^−1^) and low electrode potential (−3.04V vs. SHE) [1,2,3,4]. Nevertheless, several general obstacles still exist for lithium metal anodes. Firstly, the lithium dendrite growth, which results from uneven Li^+^ deposition, would lead to short circuits and other hazards. In extreme cases, batteries may burn or even explode due to thermal runaway caused by short circuits. In addition, the formation of dead lithium caused by the fracture of lithium dendrites can also lead to the irreversible degradation of electrode capacity. Secondly, the lithium metal anode would be corroded when it comes into contact with an electrolyte directly, leading to irreversible capacity loss and low Coulombic efficiency. Thirdly, the inhomogeneous Li^+^ deposition during the lithium plating/stripping process would lead to severe volume change and electrode pulverization [5,6,7,8,9,10].

According to the obstacles mentioned above, two different kinds of strategies have been employed to relieve lithium dendrite growth and capacity reduction. On one hand, researchers focus on surface modification by constructing an artificial solid electrolyte interface (SEI) layer between the lithium anode and electrolyte. These strategies usually include in situ growing a complete, stable, and uniform SEI with different electrolyte additives and ex situ coating a robust, flexible SEI on the surface of the lithium metal anode [6,11,12,13]. On the other hand, many efforts are also concentrated on three-dimensional (3D) conductive matrices as current collectors, which is easier for applications and considered a more promising way. Furthermore, the 3D structure can reduce local current density and accommodate the volume change during the plating/stripping process [14,15,16]. The above strategies are proven to be effective in regulating lithium deposition behavior, but the root problem of lithium dendrite growth still exists. To solve this problem, some lithiophilic active sites were used to induce the nucleation of Li^+^. So far, metal oxides, metal sulfides, metal bromides [17], and metal phosphates [18] have been proposed to obtain this property, and these materials often include Cu [19,20,21], Co [22,23,24,25], Ni [26,27,28,29], Zn [30,31,32,33,34], Fe [35,36], Sn [37,38,39], Ag [40,41], Mo [42], Al [43], Au [34,44], Mn [45], Ge [46], In, Bi, As [47], and so on. Recently, Tang’s group [48] proposed a dendrite-free Li-Co_3_O_4_/NF composite anode fabricated through the thermal infusion method between molten Li and 3D porous nickel foam (NF) decorated with lithiophilic Co_3_O_4_ nanosheet arrays. Kang’s group [18] reported a lightweight 3D nanowire network with a phosphidation gradient used as the current collector of the anode, which can balance the lithiophilicity with the conductivity of the electrode. Moreover, Shi’s group [49] proposed a composite architecture for dendrite-free lithium metal anodes through a facile electrochemical co-deposition technology that includes a 3D lithiophobic phase (Cu) and lithiophilic phase (Zn or Sn). These lithiophilic active sites are easy to react with Li^+^, which can reduce the Li^+^ nucleation overpotential. It determines the inherent normative deposition way of the lithium metal followed by nucleation and growth consecutively rather than the irregular growth way of the lithium dendrites. The works mentioned above prove that the lithiophilic modification of the current collector is an effective strategy for reducing nucleation overpotential and inhibiting lithium dendrite growth. Meanwhile, the development and application of in situ modification methods avoid the use of complex coating processes and enable them to be applied to three-dimensional current collectors.

In this work, a novel 3D lithiophilic architecture decorated with Ni_3_S_2_ nanoparticles and LPP on nickel foams (NFs) was proposed. Ni_3_S_2_ arrays on the surface of NF prepared through a facile hydrothermal method can induce the nucleation of Li^+^, thus achieving a uniform Li^+^ deposition. We selected NF as the basic material, which can not only accommodate the lithium volume expansion but can also reduce the local current densities, thus inhibiting the lithium dendrite growth. In addition, the lithiophilic LPP layer was also utilized to accelerate Li^+^ diffusion. Benefitting from the above advantages of the synergistic effect of lithiophilic LPP and Ni_3_S_2_ as well as NF, the 3D lithiophilic LPP-Ni_3_S_2_@Ni-Li anode presents very low overpotential (less than 0.02 V) and high Coulombic efficiency (99.60% for 200 cycles) at a current density of 1 mA cm^−2^. Full cells with LiFePO_4_ as the cathode also exhibit a promising electrochemical performance with over 76.78% capacity retention for 200 cycles at 1 C.

## 2. Materials and Methods

### 2.1. Preparation of Ni_3_S_2_@Ni Electrode

Nickel foam (NF) was firstly washed, respectively, by ethanol, 2 M HCl solution, and deionized water with ultrasonication to remove the surface oxide and oil ester impurities. Then, the NF was cut into the proper size and put into a breaker with the solution required for cleaning, then the breaker was transferred into an ultrasonic cleaner for 15 min. After cleaning with deionized water, the solution was replaced and the above steps were repeated. Next, NF was naturally air-dried and cut into 4 × 5 cm rectangular pieces. After that, cleaned NF was transferred to a 100 mL Teflon-lined autoclave that was filled with 70 mL of 0.2 M Na_2_S_2_O_3_ aqueous solution, and the Teflon-lined autoclave was transferred to a Muffle furnace at 120 °C for 4 h with a heating rate of 10 °C min^−1^. After the autoclave cooled down to room temperature, the prepared Ni_3_S_2_@Ni was brought out and washed with deionized water three times and naturally air-dried in a fuming cupboard.

### 2.2. Preparation of LPP-Ni_3_S_2_@Ni Electrode

The LPP is a kind of copolymer synthesized from lithium 4-styrenesulfonyl (trifluoromethylsulfonyl) imide (LiSTFSI), pentaerythritol tetrakis (3-mercaptopropionate) (PTMP), and pentaerythritol tetraacrylate (PETA), which is, therefore, denoted as LPP. The detailed preparation process of LPP can be referred to in our group’s previous work [9]. After obtaining a 1 wt% LPP precursor solution, 40 μL of 1 wt% LPP precursor solution dispersion was dropped on one piece of the Ni_3_S_2_@Ni electrode (1.13 cm^2^) and fully infiltrated. The LPP-Ni_3_S_2_@Ni electrode was generated after UV light irradiation and air-dried.

### 2.3. Material Characterization

X-ray powder diffraction (XRD) patterns were performed on an X-ray diffractometer (Empyrean, Malvern Panalytical, Shanghai, China, Cu-Kα, λ = 0.154 nm) at the 2θ range of 10–80°. Scanning electron microscope (SEM) images and energy-dispersive X-ray spectroscopy (EDS) element-mapping images were observed on a thermal field emission environmental SEM EDS EBSD. Surface species of the samples were characterized via X-ray photoelectron spectroscopy (XPS, ESCA, Nexsa, Al-Kα, 1468.6 eV).

### 2.4. Electrochemical Measurements

All cells were assembled to CR2032-type coin cells in an Ar-filled glove box, in which Celgard 2500 was used as the separator and ether-based electrolyte with 1 M lithium bis (trifluoromethanesulfonyl) imide (LiTFSI) in 1,3-dioxolane (DOL) and 1,2-dimethoxyethane (DME) (1:1 *v*/*v*) with 2% LiNO_3_ additive as the electrolyte; the amount of the electrolyte used was 50 μL in each cell. For half-cells, the prepared LPP-Ni_3_S_2_@Ni electrodes were directly employed as working electrodes, while lithium was used as the counter electrode. To test Coulombic efficiency, the cell was firstly cycled between 0 and 1 V at 50 μA for 5 cycles for activation, and then, 1 mAh cm^−2^ of lithium was deposited on the working electrode and stripped away until the voltage reached 1 V at 1 mA cm^−2^. For symmetric cells, the LPP-Ni_3_S_2_@Ni was first deposited with 4 mAh cm^−2^ of lithium in a half-cell, and lithium acted as the counter electrode. The cycling performance of symmetric cells was tested at 1 mA cm^−2^ with a capacity of 1 mAh cm^−2^. For full cells, the LPP-Ni_3_S_2_@Ni was first deposited with 3 mAh cm^−2^ of lithium (mass loading: 0.778 mg cm^−2^) (because the modified active materials will sacrifice some capacity, actual lithium deposition capacity will be different from the electrochemical deposition capacity). The generated LPP-Ni_3_S_2_@Ni-Li electrode was employed as an anode of the full cells while LiFePO_4_ was employed as the cathode. The mass loading of LiFePO_4_ in the cathode was about 1.7 mg cm^−2^.

## 3. Results

**Figure 1:**
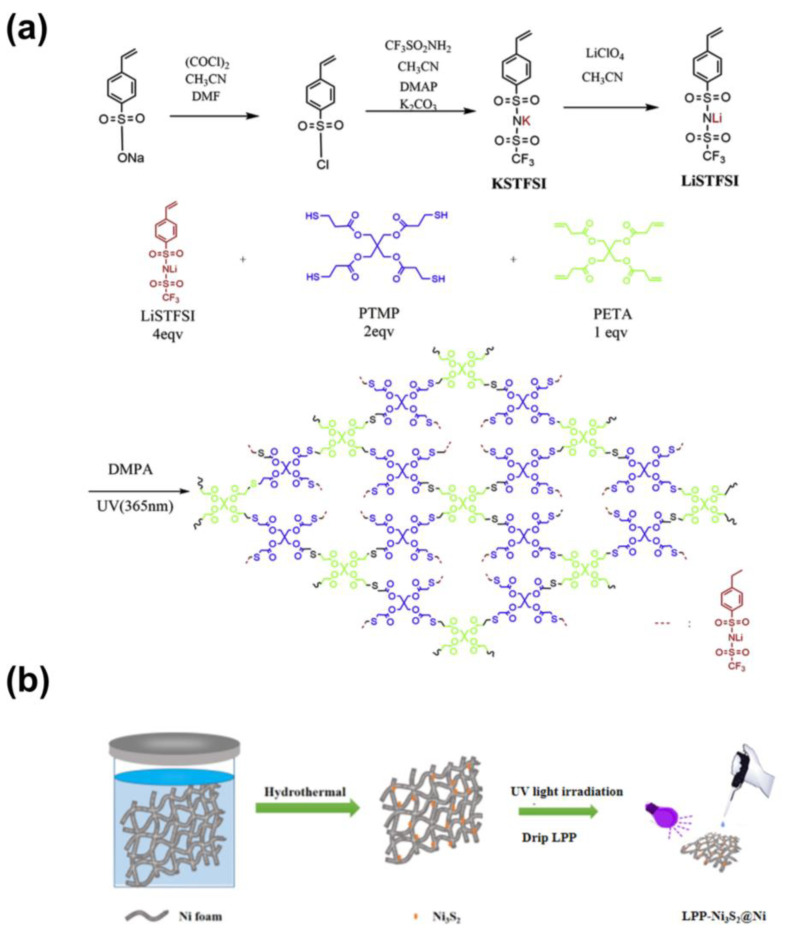
(**a**) Schematic illustration of the synthetic process of LPP. (**b**) The typical preparation process of LPP-Ni_3_S_2_@Ni.

The typical preparation process of LPP is schematically illustrated in Figure 1a. Three-dimensional porous nickel foam (NF) was firstly immersed in Na_2_S_2_O_3_ precursor to go through a facile hydrothermal process. After being washed and dried, the three-dimensional Ni_3_S_2_@Ni electrode was prepared. And that, LPP (1 wt%) was dropped onto NF and infiltrated completely. After drying, the three-dimensional LPP-Ni_3_S_2_@Ni electrode with lithiophilic architecture was prepared.

**Figure 2:**
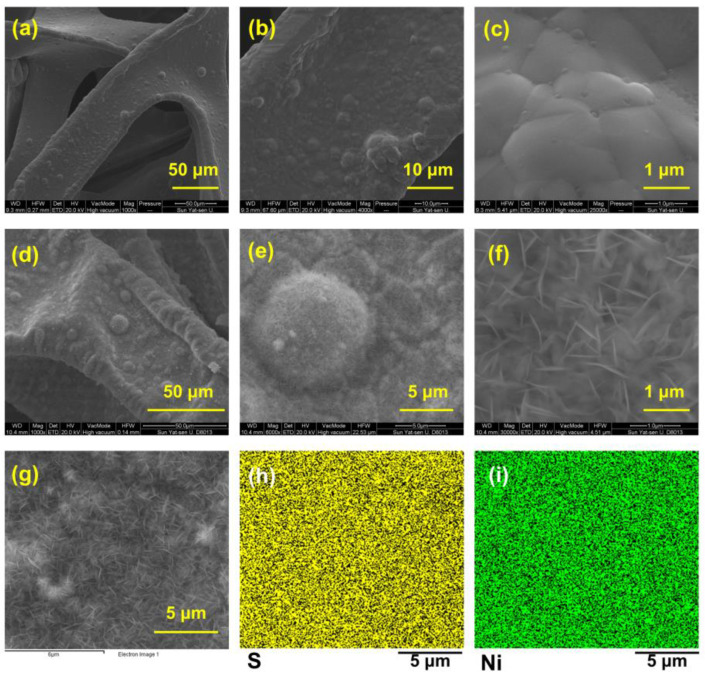
SEM images of bare nickel foam current collector (**a**–**c**) and Ni_3_S_2_@Ni current collector (**d**–**f**). Mapping of Ni_3_S_2_@Ni current collector (**g**–**i**).

The scanning electron microscope (SEM) images in Figure 2a–f show the surface morphology of bare NF (Figure 2a–c) and Ni_3_S_2_@Ni (Figure 2d–f). We can see that, compared with the smooth surface of the bare NF, the Ni_3_S_2_@Ni electrode surface was covered with sheet-like nanoparticles, which were thought to be Ni_3_S_2_. In order to confirm that the nanosheet particles are nickel sulfide, the energy spectrum analysis was performed on the area where the nanosheet was produced. As shown in Figure 2h,i, it was found that both the Ni element and S element were evenly distributed on the surface. The sulfurized surface of Ni_3_S_2_@Ni was confirmed immediately via energy-dispersive X-ray spectrometer mapping, which entails the successful preparation of Ni_3_S_2_ on the surface of Ni_3_S_2_@Ni electrode. In addition, the content of different elements in mapping spectra is displayed in Table 1. It can be found that in addition to the formation of nickel sulfide, a part of nickel oxide is also produced on the surface of NF.

**Figure 3:**
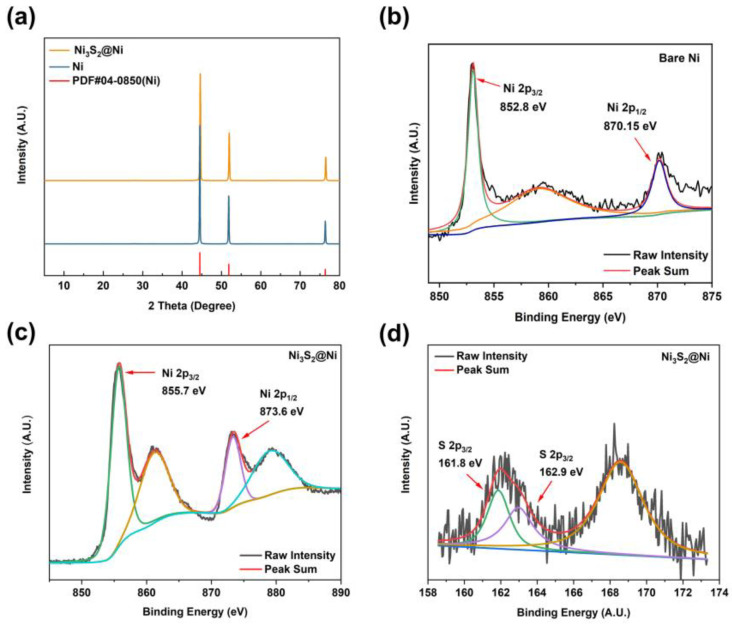
(**a**) XRD pattern of Ni_3_S_2_@Ni current collector. XPS spectra of bare Ni (**b**) and Ni_3_S_2_@Ni current collector (**c**,**d**).

To further characterize the successful preparation of Ni_3_S_2_@Ni, XRD was carried out to obtain their crystal structure information (Figure 3a). By comparing the diffraction peaks of Ni and Ni_3_S_2_@Ni with the standard PDF data, three distinct diffraction peaks can be observed. They are, respectively, located at 44.21° (111), 51.51° (200), and 75.84° (220). However, the XRD pattern of the Ni_3_S_2_@Ni electrode only has obvious characteristic peaks of Ni. The peaks of Ni_3_S_2_ are not obvious in the spectrogram, which means the loading of Ni_3_S_2_ on the surface of NF is controlled at a very low level and the modification reaction occurs solely on the ultrathin surface of NF without damaging the substrate. In order to confirm that the modifier is Ni_3_S_2_, XPS was used consequently, and the results are shown in Figure 3b–d. In the Ni 2p spectrum, two main peaks at 855.7 eV and 873.6 eV can be assigned to Ni 2p 3/2 and Ni 2p 1/2, respectively. The binding energy of metallic Ni is 852.8 eV (2p 3/2) and 870.15 eV (2p 1/2). As Ni_3_S_2_ was successfully synthesized, the electron cloud density near the Ni element decreased due to the formation of ionic bonds, resulting in a weakened shielding effect. Therefore, valence electrons around the Ni element obtained a higher binding energy of 855.7 eV (2p 3/2) and 873.6 eV (2p 1/2). And, in the S 2p spectrum, peaks at 161.8 eV and 162.9 eV can be assigned to S 2p 3/2. The binding energy of S 2p 3/2 in Ni_3_S_2_ is reported to be 163.0 eV, which is consistent with our XPS results. As for the peak at 161.8 eV, this may be ascribed to the formation of NiS (162.1 eV). In a word, the spectra of Ni 2p and S 2p prove that the modifier on NF is Ni_3_S_2_.

**Figure 4:**
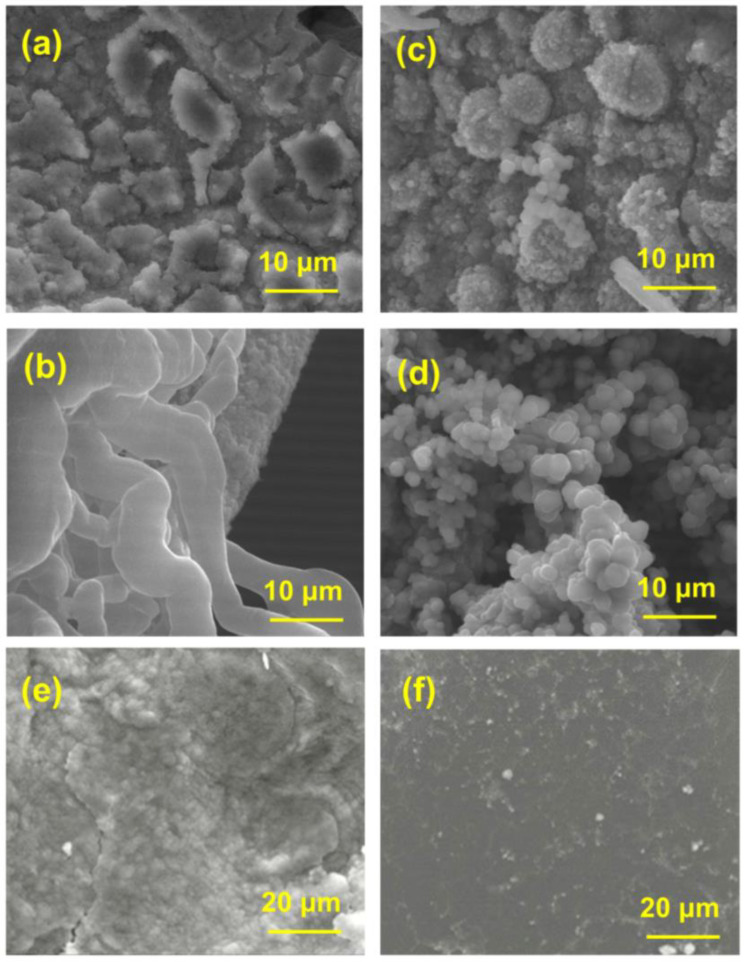
SEM images of Ni-Li electrode with (**a**) 0.5 mAh cm^−2^ and (**b**) 2 mAh cm^−2^ lithium deposition. SEM images of Ni_3_S_2_@Ni-Li electrode with (**c**) 0.5 mAh cm^−2^ and (**d**) 2 mAh cm^−2^. SEM images of (**e**) Ni-Li and (**f**) LPP-Ni_3_S_2_@Ni-Li electrode after 500 cycles.

Half-cells were constructed to characterize the surface morphology of the anode after lithium deposition. Firstly, different loadings of lithium (0.5 mAh cm^−2^ and 2 mAh cm^−2^) were deposited on NF and Ni_3_S_2_@Ni, and their SEM images are shown in Figure 4a–d. Figure 4a shows that Li^+^ is deposited irregularly on bare NF substrate. When deposition loading increases to 2 mAh cm^−2^, a dendritic morphology appears easily (Figure 4b). Different from bare NF, the lithium deposited on the surface of Ni_3_S_2_@Ni was more regular (Figure 4c). There was no obvious lithium dendrite even at a high loading (2 mAh cm^−2^) of lithium deposition (Figure 4d). It was considered that the Ni_3_S_2_ can induce the nucleation of Li^+^ and the process of deposition is nucleation followed by growth. Therefore, it can be speculated that Li^+^ preferentially nucleates at the “lithophilic” active site [50] of Ni_3_S_2_, which may be because Ni_3_S_2_ can reduce the nucleation overpotential of Li^+^. Thus, the deposition of lithium can be controlled, which helps to solve the dendritic problems of the lithium anode. Surface SEM images of composite anodes in symmetric cells are shown in Figure 4e,f. It can be seen that for the Ni-Li anode after 500 cycles, there is an uneven surface with a loose structure and huge cracks (Figure 4e). Meanwhile, for the LPP-Ni_3_S_2_@Ni-Li anode, as shown in Figure 4f, there is a flat surface with no obvious cracks and dendritic growth after 500 cycles, indicating that the LPP-Ni_3_S_2_ modified on the NF current collector can guide the deposition of Li^+^.

**Figure 5:**
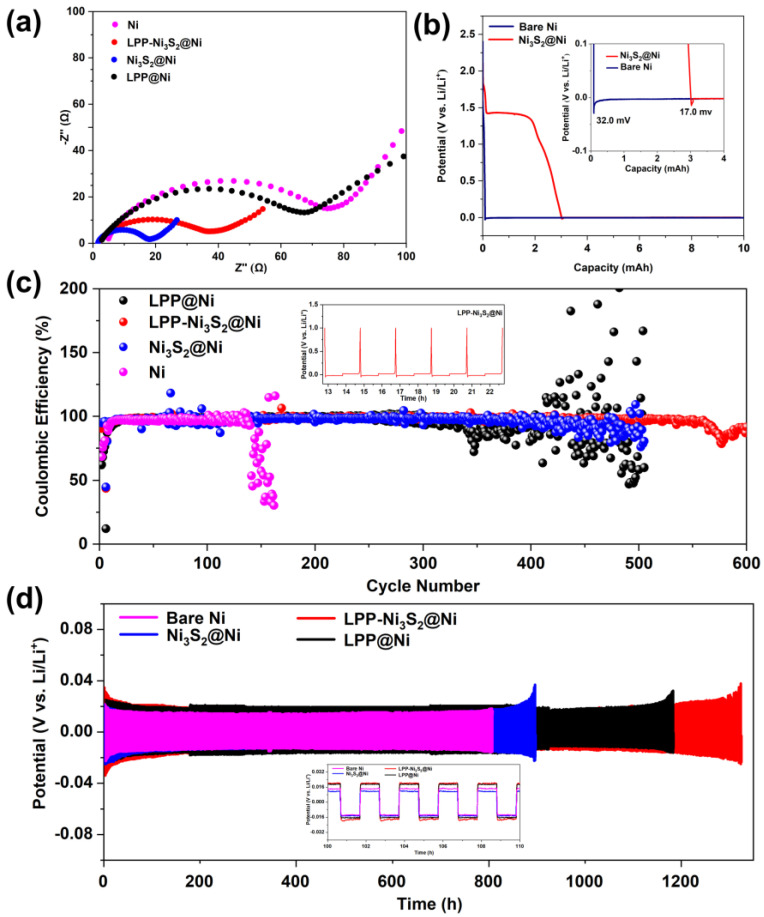
(**a**) EIS for half-cells of Ni|Li, LPP@Ni|Li, Ni_3_S_2_@Ni|Li, and LPP-Ni_3_S_2_@Ni|Li. (**b**) Nucleation overpotential of bare Ni and Ni_3_S_2_@Ni. (**c**) Coulombic efficiency of half-cells assembled with bare Ni, LPP@Ni, Ni_3_S_2_@Ni, and LPP-Ni_3_S_2_@Ni. (**d**) Voltage-time profiles of Ni-Li|Li, LPP@Ni-Li|Li, Ni_3_S_2_@Ni-Li|Li, and LPP-Ni_3_S_2_@Ni-Li|Li symmetric cells.

Bare Ni|Li, LPP@Ni|Li, Ni_3_S_2_@Ni|Li, and LPP-Ni_3_S_2_@Ni|Li half-cells were assembled, and EIS tests were performed. The Nyquist curves are shown in Figure 5a. It can be seen from the curves that after the modification of LPP and Ni_3_S_2_, the interfacial impedance of the cell is significantly reduced from 78 Ω to 35 Ω. The bulk resistance (R_b_) of Ni decreased from 5 Ω to 2 Ω, benefiting from a larger specific surface area and more abundant active sites. Because R_b_ is substantially determined via the conduction of Li^+^ in non-interface regions, the decrease in R_b_ can also prove that the transport rate of Li^+^ becomes higher. Since LPP has an anion-fixed network with a cross-linked structure, it can effectively limit the migration of anions and realize single-ion conduction dominated by Li^+^. In addition, compared with the in situ generated SEI, LPP can act as an isolating barrier to inhibit the reaction between electrolyte and active fresh lithium metal, thereby improving the Coulombic efficiency of the lithium metal anode and reducing the generation of dead lithium. Meanwhile, LPP with a cross-linked network structure has a certain strength, which can hinder the growth of lithium dendrites and prevent the separator from being punctured, improving the safety of the batteries. The deposition profiles of Ni and Ni_3_S_2_@Ni at a constant current (0.05 mA cm^−2^) are shown in Figure 5b. Firstly, 1 mAh cm^−2^ of lithium was deposited on the composite current collector. According to reference [34], the difference between the flat part of the voltage plateau and the bottom of the voltage drop is used to define the nucleation overpotential. It can be seen from Figure 5b that the nucleation overpotential of Ni_3_S_2_-modified NF (Ni_3_S_2_@Ni, 17.0 mV) is lower than that of the bare NF (32.0 mV), indicating that the presence of Ni_3_S_2_ can indeed decrease the nucleation overpotential of Li^+^. Furthermore, the Ni_3_S_2_ has been reported with “lithophilic” characteristics [50]. Figure 5c shows the Coulombic efficiency of the half-cells of bare NF, LPP@Ni, Ni_3_S_2_@Ni, and LPP-Ni_3_S_2_@Ni, assembled with lithium as a counter electrode. It can be seen that the half-cell assembled with LPP-Ni_3_S_2_@Ni showed better stability for more than 500 cycles. The Coulombic efficiency of half-cells assembled with Ni_3_S_2_@Ni and LPP@Ni decreases after 300 cycles, and the half-cells assembled with bare NF show the lowest Coulombic efficiency, which decreased at about the 120th cycle. From the point of view of Coulombic efficiency, the LPP-Ni_3_S_2_@Ni showed a better charging and discharging performance. The time–voltage diagrams of Ni-Li, LPP@Ni-Li, Ni_3_S_2_@Ni-Li, and LPP-Ni_3_S_2_@Ni-Li utilized in symmetrical cells are shown in Figure 5d. It can be seen that the symmetric cell composed of bare NF has a significant increase in polarization before 900 h, while LPP@Ni-Li|Li, Ni_3_S_2_@Ni-Li|Li, and LPP-Ni_3_S_2_@ Ni-Li|Li symmetric cells evidently show better long-cycle stability. Among them, Ni_3_S_2_@Ni-Li|Li symmetric cells can cycle stably for about 1100 h, LPP@Ni-Li|Li can cycle stably for 1300 h, and LPP-Ni_3_S_2_@Ni-Li|Li symmetric cells show the most superior cycle stability, which is up to 1300 h.

A summary of recent works is shown in Table 2. It can be found that in general, in plating/stripping experiments in symmetric cells, the LPP-Ni_3_S_2_@Ni-Li composite anode has a relatively more stable performance and longer lifespan. Both of the Li-Co_3_O_4_/NF [48] and AuLi_3_@Ni foams [34], which used NF as a 3D current collector, show significant plating/stripping stability. As for interfacial resistance, the LPP-Ni_3_S_2_@Ni-Li can also achieve an average level even though the usage of polymer-based LPP could sacrifice part of the interface ion transport rate. Meanwhile, the LPP can effectively protect the lithium metal anode surface from being destroyed by continuous side reactions between lithium and electrolyte.

**Figure 6:**
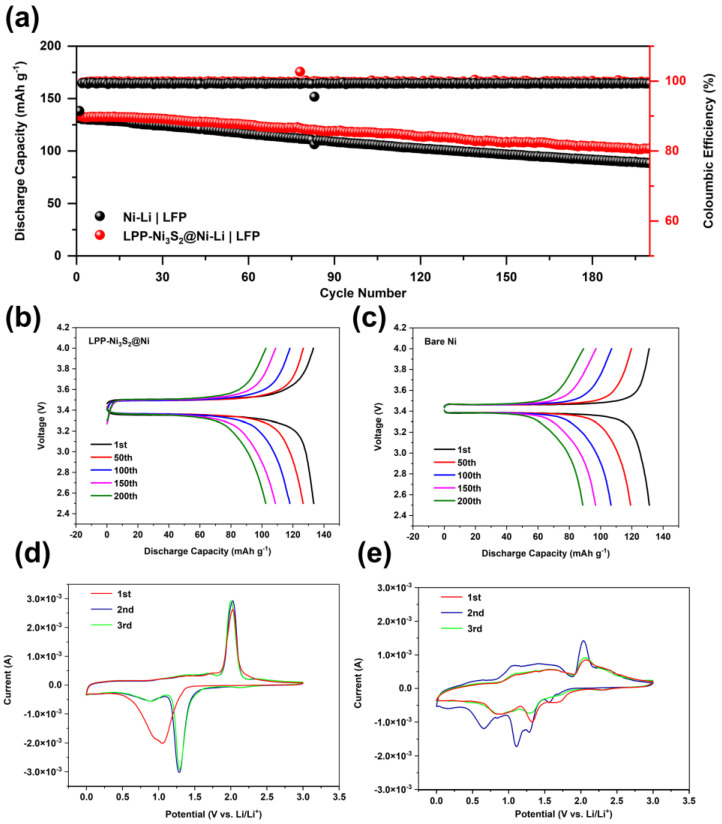
(**a**) Cycle stability for full cells of LPP-Ni_3_S_2_@Ni-Li|LFP and Ni-Li|LFP. Charge–discharge profiles of (**b**) LPP-Ni_3_S_2_@Ni-Li|LFP and (**c**) Ni-Li|LFP cells. Cyclic Voltammetry Curves of LPP-Ni_3_S_2_@Ni-Li (**d**) and Ni_3_S_2_@Ni-Li (**e**) half-cells.

For full cells, 3 mAh cm^−2^ of lithium was first deposited on LPP-Ni_3_S_2_@Ni current collector to fabricate LPP-Ni_3_S_2_@Ni-Li a composite lithium metal anode, and an electrode with LiFePO_4_ (1.7 mg cm^−2^) was employed as a cathode. Meanwhile, the bare NF was treated in the same way as LPP-Ni_3_S_2_@Ni and tested as the reference group. The cycle stability performance of the full cell is shown in Figure 6a–c. The cycle performance of the LPP-Ni_3_S_2_@Ni-Li composite anode is better than that of bare Ni-Li. This is because the lithiophilic structure of LPP-Ni_3_S_2_@Ni can guide Li^+^ to nucleate and deposit uniformly on the surface; meanwhile, the LPP with a crosslinked structure can further help regulate the Li^+^ flux and the deposition of lithium. Furthermore, the reversibility and lithium plating/stripping stability are also improved via the lithiophilic LPP layer, which is manifested through Cyclic Voltammetry Curves in Figure 6d,e. Furthermore, the three-dimensional NF can decrease the local current density and help uniformly disperse electron distribution, thereby further promoting the uniformity of the lithium deposition process and improving battery performance. The Coulombic efficiency of bare Ni-Li|LFP cells is lower than that of LPP-Ni_3_S_2_@Ni-Li|LFP cells and was maintained at 99.20% and 99.60%, respectively. For capacity, LPP-Ni_3_S_2_@Ni-Li|LFP shows higher initial specific capacity (133.5 mAh g^−1^) than Ni-Li|LFP (131.2 mAh g^−1^), and the capacity remains 76.78% and 67.53%, respectively, after 200 cycles. The details of the charge and discharge curves of LPP-Ni_3_S_2_@Ni-Li|LFP batteries and bare NF-Li|LFP batteries are shown in Figure 6b,c. We can find that the cell modified with the lithiophilic architecture has greater polarization but its capacity retention rate is higher.

It can be found that the in situ generation of sulfides is an effective method for the lithiophilic modification of the current collector surface, which can help reduce the energy barrier of Li^+^ deposition as well as the nucleation overpotential, guiding the uniform lithium deposition. The application of three-dimensional current collector NF can also effectively improve the plating/stripping cycle stability of composite lithium metal anodes. This is due to its ability to restrict the growth of lithium dendrites within its three-dimensional framework structure, reducing the risk of short circuits caused by lithium dendrites piercing the separator. In addition, the introduction of LPP also has a significant inhibitory effect on the growth of lithium dendrites. At the same time, the protection of the lithium metal anode surface via LPP greatly improves the Coulombic efficiency and cycle reversibility. Under the synergistic effect of the methods mentioned above, LPP-Ni_3_S_2_@Ni-Li successfully inhibits the growth of lithium dendrites and achieves a better electrochemical performance.

## 4. Conclusions

A lithiophilic architecture LPP-Ni_3_S_2_@Ni anode, in which the lithiophilic component nickel sulfide can be grown in situ on the current collector through a simple hydrothermal reaction, is reported to be able to guide the nucleation and deposition of lithium in this paper. The lithiophilic component LPP can be obtained via photoinitiation and drip-coated on the current collector to help standardize lithium deposition. Furthermore, we selected three-dimensional nickel foam as the current collector to reduce current density, contain volume expansion, and reduce polarization. As a result, the LPP-Ni_3_S_2_@Ni-Li anode with a lithiophilic interface structure exhibits enhanced electrochemical performance in symmetric cells with lower overpotential (less than 0.02 V) and longer cycling time (about 1300 h). In addition, the prepared 3D LPP-Ni_3_S_2_@Ni gives a high Coulombic efficiency of 99.60% for 200 cycles at 1 mA cm^−2^. Full cells with LiFePO_4_ as the cathode also exhibit a promising electrochemical performance with 76.78% capacity retention for 200 cycles at 1 C. The prepared 3D LPP-Ni_3_S_2_@Ni-Li maintains a reversible capacity of 133.3 mAh g^−1^ with a stable CE of 99.60% and a capacity retention of 76.78% after 200 cycles, indicating its great potential as a lithium metal anode. In summary, the lithiophilic architecture’s design offers avenues for exploring effective solutions to the lithium dendrite issue.

## Figures and Tables

**Table 1:** Percentage of elements on Ni_3_S_2_@Ni surface obtained via EDS.

Element	Weight (%)	Atomic (%)
C	5.72	15.08
O	17.81	35.24
Na	0.53	0.73
S	17.87	17.64
Ni	58.07	31.31
Totals	100	100

**Table 2:** Summary of electrochemical performance of similar works.

Sample	Current Density (mA cm^−2^)	Capacity Density (mAh cm^−2^)	Stability for Symmetric Cells (h)	R_ct_ (Ω)	Ref.
GZCNT-Li	1	1	1100	75	[51]
Li-rGO	1	1	222	32	[52]
Li-Co_3_O_4_/NF	3	1	1000	10	[48]
Li_13_In_3_|Li	2	2	1200	NA	[47]
Li-Mn/G	2	1	300	30	[45]
AuLi_3_@Ni foam	0.5	1	720	13	[34]
LPP-Ni_3_S_2_@Ni	1	1	1300	35	This Work

## Data Availability

Data are contained within the article.
